# Quantitative imaging of fibrotic and morphological changes in liver of non-alcoholic steatohepatitis (NASH) model mice by second harmonic generation (SHG) and auto-fluorescence (AF) imaging using two-photon excitation microscopy (TPEM)

**DOI:** 10.1016/j.bbrep.2016.09.010

**Published:** 2016-09-25

**Authors:** Shin Yamamoto, Yusuke Oshima, Takashi Saitou, Takao Watanabe, Teruki Miyake, Osamu Yoshida, Yoshio Tokumoto, Masanori Abe, Bunzo Matsuura, Yoichi Hiasa, Takeshi Imamura

**Affiliations:** a Department of Molecular Medicine for Pathogenesis, Ehime University Graduate School of Medicine, Japan; b Department of Gastroenterology and Metabiology, Ehime University Graduate School of Medicine, Japan; c Division of Bio-imaging, Proteo-Science Center, Ehime University, Japan; d Translational Research Center, Ehime University Hospital, Japan; e Core Research for Evolutional Science and Technology (CREST), Japan Science and Technology Agency (JST), Japan; f Department of Lifestyle-related Medicine and Endocrinology, Ehime University Graduate School of Medicine, Japan

**Keywords:** NASH, non-alcoholic steatohepatitis, SHG, second harmonic generation, AF, auto-fluorescence, TPEM, two-photon excitation microscopy, Fluorescence imaging, Fibrosis, Collagen, NASH, SHG, Two-photon excitation microscopy

## Abstract

Non-alcoholic steatohepatitis (NASH) is a common liver disorder caused by fatty liver. Because NASH is associated with fibrotic and morphological changes in liver tissue, a direct imaging technique is required for accurate staging of liver tissue. For this purpose, in this study we took advantage of two label-free optical imaging techniques, second harmonic generation (SHG) and auto-fluorescence (AF), using two-photon excitation microscopy (TPEM). Three-dimensional *ex vivo* imaging of tissues from NASH model mice, followed by image processing, revealed that SHG and AF are sufficient to quantitatively characterize the hepatic capsule at an early stage and parenchymal morphologies associated with liver disease progression, respectively.

## Highlights

• Combination of two label-free optical imaging techniques for the evaluation of liver disease is proposed.
• SHG is sufficient to quantitatively characterize the hepatic capsule at an early stage of NASH.
• Auto-fluorescence is useful to evaluate the parenchymal morphologies associated with liver disease progression.

## 1 Introduction

Hepatocellular carcinoma and liver cirrhosis are common chronic liver diseases primarily caused by hepatitis viral infection. Recently, the rate of virus-related hepatocellular carcinoma has decreased because of improvements in antiviral therapeutics. On the other hand, the incidence of non-alcoholic fatty liver disease (NAFLD) and its progressive form, non-alcoholic steatohepatitis (NASH), has been increasing, and NASH may be the leading cause of alcoholic- and virus-independent cirrhosis and hepatocellular carcinoma in developing countries [1], [2]. Because NASH causes not only liver cirrhosis and hepatocellular carcinoma but also other lethal disorders such as cardiovascular disease, it is essential to accurately diagnose NASH to prevent the progression of fibrosis [2], [3], [4].

Various markers of fibrosis, including platelet count, hyaluronic acid, and type 4 collagen, have been used to diagnose NASH. In addition, elastography is used to evaluate the stiffness of liver tissue, which may reflect the degree of fibrosis [5], [6]. However, definitive diagnosis of NASH requires histopathological examination of liver biopsy samples. In such examinations, steatosis, lobular inflammation, and fibrosis are important pathological findings that contribute to a diagnosis of NASH. Early detection of these pathological findings in patients with NASH is difficult, both because there is a sampling bias and because the diagnosis depends on the experience of the pathologist [7].

Optical imaging techniques make it possible to visualize structure, morphology, and molecular distribution *in vivo* at the cellular level. In particular, the cutting-edge technique of two-photon excitation microscopy (TPEM) is a promising tool for diagnosis of several types of disorders, e.g., cancer, vascular disease, fibrotic diseases [8], [9], [10]. Fibrosis is generally characterized by accumulation of collagen, which can be observed directly without staining due to a nonlinear optical phenomenon called second harmonic generation (SHG). In the nonlinear interaction between incident photons and molecules in tissue, non-centrosymmetric molecules such as collagen, myosin, and tubulin generate light with half the wavelength of the excitation source. In the last two decades, SHG imaging has been used not only for characterization of normal collagenous tissues [11], [12], but also for diagnostic evaluation of various fibrotic diseases [13], [14], [15]. Recently, several groups applied SHG imaging to staging of liver fibrosis in human biopsied samples [16], [17]. Their studies, which analyzed methods for fibrosis scoring in SHG images of fibrotic collagen from histological slice samples, demonstrated that SHG scoring of fibrillar collagen deposits correlates well with conventional scoring of liver fibrosis by pathologists, e.g., Metavir scoring,. However, liver tissue slices can reveal changes occurring in the liver parenchyma, but not those in the liver surface. Because the liver capsule provides a large amount of collagen fibrils, observation of liver fibrosis in that layer provides a new opportunity for evaluation of fibrosis. Moreover, NASH is diagnosed by fibrosis, concomitantly with hepatic fat accumulation and hepatocellular injury. Therefore, to fully exploit the potential of non-staining imaging by TPEM, it would be desirable to develop a new imaging target that is compatible with SHG imaging and suitable for characterization of liver steatosis in hepatic parenchyma.

Here we demonstrate SHG and auto-fluorescence (AF) *ex vivo* imaging of liver tissues in NASH model mice. Using three-dimensional (3D) SHG imaging, image processing by maximum intensity projection (MIP), and statistical analyses, we successfully characterized the fibrotic changes in hepatic capsule of NASH model mice. In addition, we investigated the use of auto-fluorescence (AF) imaging in the model. Applying the same image processing procedure to AF images, we could detect the morphological characteristics of hepatic parenchyma. The direct and quantitative SHG and AF imaging method presented in this study could be applied to diagnosis of NASH and other fibrotic diseases.

## 2 Materials and methods

### 2.1 Animals

STAM mice, a commercially available NASH–cirrhosis–hepatocarcinogenesis model, were purchased from Stelic Institute & Co., Inc. (Tokyo, Japan). STAM mice were established as described previously [18]. In brief, 2-d-old male C57BL⁄6J mice were treated subcutaneously with streptozotocin (200 μg/mouse) and fed a high-fat diet HFD-32 (CLEA Japan, Tokyo, Japan) from the age of 4 weeks. Because this model mouse exhibits fatty liver at 5 weeks of age and fibrosis at 9 weeks, we selected 6- and 9-week-old mice for experiments.

A total of nine STAM mice were prepared as a model for NASH; these mice were sacrificed and analyzed at the age of 6 weeks (n =4) or 9 weeks (n =5). Liver was harvested under deep anesthesia, and the mice were euthanized by intraperitoneal (i.p.) injection of a lethal dose of anesthetic (sodium pentobarbital, >100 mg/kg body weight; Kyoritsu Seiyaku Co., Tokyo, Japan). C57BL⁄6J mice (CLEA Japan) used as controls were fed normal food and analyzed at the age of 6 weeks (n=3) or 9 weeks (n=3). All animals were kept in normal cages, allowed free access to water and food, and maintained in a temperature-controlled specific pathogen–free animal facility. All experiments and procedures were approved by the Ehime University Animal Research Committee (#05-RE-1–16).

### 2.2 Two-photon excitation microscopy (TPEM)

Image acquisition of SHG and AF signals from the middle lobe of the liver was performed using an upright two-photon excitation microscopy (TPEM) system (A1RMP, Nikon, Tokyo, Japan) as previously described [8], [19], [20]. In brief, excised livers were embedded in agarose gel, and the liver mid-ventral aspect was exposed under the water-immersion objective lens (CFI75 Apo 25xW MP, numerical aperture: 1.1, Nikon) of the TPEM system. An excitation wavelength of 950 nm (Ti:Sapphire laser oscillator; wavelength, 680–950 nm; repetition rate, 80 MHz; pulse width, 70 fs; MaiTai eHP, Spectra-Physics, Santa Clara, CA, USA) was employed for both SHG and AF image acquisition.

SHG signals were acquired using a short-pass filter at 492 nm. A dichroic mirror at 560 nm and an emission filter at 525/50 nm (center wavelength / bandwidth) were used for simultaneous multicolor imaging. Images were obtained from the surface of the liver tissue to the deep portion (~100 µm in depth) and stored as Z-stack image sequences (step size, 1 µm along the z-axis). Images were 512×512 pixels, and pixel size was 0.48 µm. Image acquisition time was 1 frame/sec in all experiments. Simple image processing, e.g., merging color channels and three-dimensional (3D) rendering, was performed using the NIS-Elements ver. 4.0 software package (Nikon).

### 2.3 Histological analysis of liver

The left lobe of the liver was fixed overnight with 4% paraformaldehyde in phosphate-buffered saline (PBS) and embedded in paraffin. Five-micrometer sections were prepared and deparaffinized with xylene. For general histological analysis, hematoxylin and eosin (HE) staining was performed. The degree of liver fibrosis was assessed by Elastica Masson–Goldner (EMG) staining.

### 2.4 Quantitative image analysis

For analysis of SHG images, the Z-stack image sequence was converted to the maximum intensity projection (MIP) using the NIS-Elements software. Briefly, for each X–Y coordinate, the highest pixel intensity among all images in the Z-stack was extracted, and a new X–Y image was generated using these maximum values. The resultant single-layer images were converted to black-and-white (binary valued) images using the ‘Default’ thresholding command in the ImageJ software. The proportion of white (SHG) area in the images was also calculated using ImageJ. For analysis of AF (red) images, the Z-stack was also converted to the MIP. The resultant images were then converted to binary-valued images by arbitrarily setting the threshold value, and areas of binarized objects more than 36 pixels in size were selectively measured using ImageJ.

### 2.5 Statistical analysis

The two-sided Student's *t*-test was used as a test of statistical significance. This test was applied to comparisons of ratios of SHG areas and average areas of objects from AF (red) images. Differences were considered to be significant when *p*<0.05. These analyses were performed using the Excel software (Microsoft, Redmond, USA).

## 3 Results

### 3.1 Image-processing procedure and model mouse

To evaluate the fibrotic and morphologic changes of liver tissues in a disease model, we first developed a NASH model and used a TPEM system to perform combined SHG and AF imaging of *ex vivo* liver tissues dissected from these mice.

**Fig. 1:**
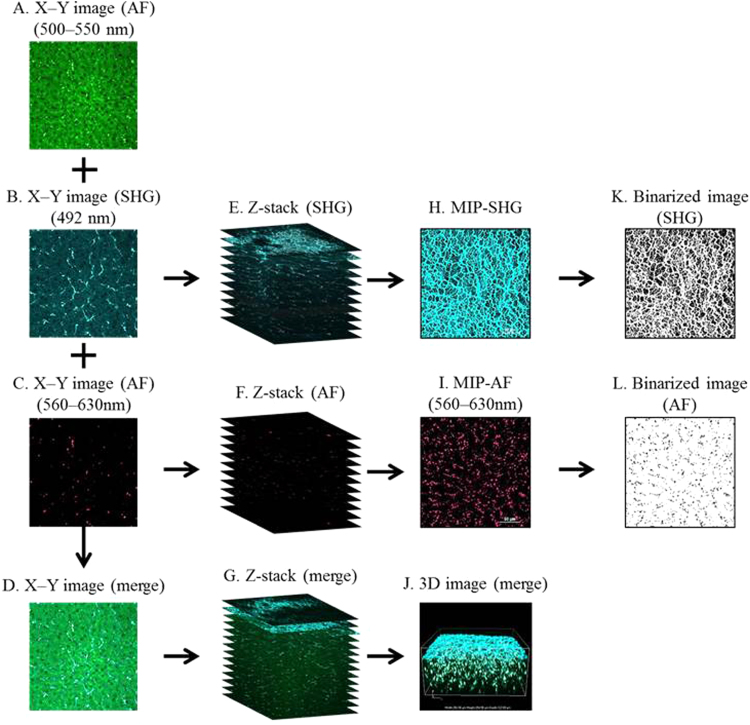
Image-processing procedure for liver tissue specimens. The left column shows X–Y images of AF (500–550 nm) (A), SHG (B), AF (560–630 nm) (C), and merge (D). The second column from the left shows Z-stacks of 2D images of SHG (E), AF (F), and merge (G). The second column from the right shows maximum intensity projection (MIP) images of SHG (H) and AF (I), and merged 3D images (J). The right column shows binarized images of (H) and (I) (K and L, respectively).

Fig. 1 demonstrates our analytical procedure for imaging and subsequent image processing (Fig. 1 A–L) using 6 weeks old and 9 weeks old NASH model mice. Two-dimensional (2D) images (X–Y images) were obtained as follows: AF, emission filter at 500–550 nm (Fig. 1A); SHG, short-pass filter at 492 nm (Fig. 1B); and AF at 560–630 nm (Fig. 1C), with excitation at 950 nm. The resultant images were merged (Fig. 1D). To quantify fibrotic and morphological changes of mouse liver tissues, we obtained maximum intensity projection (MIP) images of SHG (Fig. 1H) and AF (Fig. 1I) from Z-stacks of 2D images (Fig. 1E and F, respectively), followed by binarization of the images (Fig. 1K and L, respectively). These reconstructed images were subjected to quantitative evaluation using the ImageJ software. Merged three-dimensional (3D) images were reconstructed using the Z-stack of merged 2D images (Fig. 1G) acquired using SHG with a short-pass filter at 492 nm (cyan) and AF with emission filters at 500–550 nm (green) and 560–630 nm (red). The merged 3D images revealed structural patterns of collagen fibers and distribution patterns of AF signals in liver tissue (Fig. 1J).

For our NASH model, we used STAM® mice (Stelic Institute & Co., Inc.). First, we generated the model mice and performed histopathological analysis in comparison with control mice at the age of 6 or 9 weeks (Suppl. Fig. 1). Liver-to-body-weight were significantly higher in the STAM group (6 weeks old, 8.2±1.9%; 9 weeks old, 8.4±0.8%) than in the control group (6 weeks old; 4.93±0.8%, 9 weeks old; 4.90±0.7%) (data not shown). HE staining of liver tissue sections revealed irregular arrays of hepatic cell cord and depositions of fat droplets in specimens of NASH model mice, but not in control mice (Suppl. Fig. 1A, C, E, and G). Notably, ballooning degeneration of hepatocytes (indicated by a black arrow) was observed in 9-week-old NASH model mice (Suppl. Fig. 1G and I). In both NASH model mice and control mice, EMG staining revealed elastic fibers of connective tissue around central veins and Glisson’s capsule (Suppl. Fig. 1B, D, F, and H). In addition, mild fibrosis around the vessel (indicated with a black arrow in Suppl. Fig. 1H and J) and peripheral hepatocellular fibrosis (indicated with a black arrowhead in Suppl. Fig. 1H and J) were observed in 9-week-old NASH model mice.

### 3.2 Two- and three-dimensional reconstructed images of SHG and/or AF in liver tissues

**Fig. 2:**
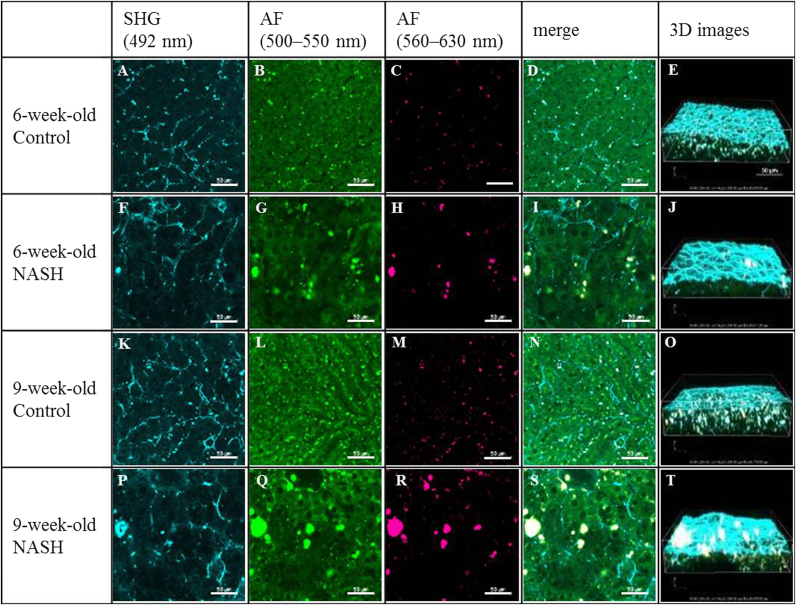
SHG, AFs, and merged X–Y images and merged 3D images in liver tissues. SHG images (A, F, K, and P), AF (500–550 nm) images (B,G, L, and Q), AF (560–630 nm) images (C, H, M, and R), and merged images (D, I, N, and S) of liver from control mice at 6 weeks (top panels), NASH model mice at 6 weeks (second panels), control mice at 9 weeks (third panels), and NASH model mice at 9 weeks (bottom panels). Three-dimensional reconstructed images of SHG and AF in liver tissues were obtained from control mice at 6 weeks of age (E), NASH model mice at 6 weeks (J), control mice at 9 weeks (O), and NASH model mice at 9 weeks (T). Scale bars, 50 µm.

Next, we obtained SHG with a short-pass filter at 492 nm (Fig. 2A, F, K, and P), AF with emission filters at 500–550 nm (Fig. 2B, G, L, and Q) and 560–630 nm (Fig. 2C, H, M, and R), and merged X–Y images (Fig. 2D, I, N, and S) from liver tissues dissected from NASH model and control mice. The SHG signal indicated that extra-cellular matrix (ECM) was distributed around hepatocytes (Fig. 2A, F, K, and P). Fibrotic changes of ECM around hepatocytes were more extensive in NASH model mice than in control mice at 9 weeks (Fig. 2K and P); this difference was less apparent at 6 weeks (Fig. 2A and F). In particular, consistent with HE staining, irregular arrays of hepatic cell cord were observed in NASH model mice, but not in control mice, at 9 weeks (Fig. 2P). Spots were observed in X–Y images of AF (500–550 nm) and AF (560–630 nm), some of which were localized identically in the two AF channels (Fig. 2B, C, G, H, L, M, Q, and R). These spots were larger and more heterogeneous in NASH model mice than in control mice. These results suggest that X–Y images of SHG partially reflect the findings of HE staining in liver tissues of NASH model mice. The fibrotic changes were apparent in 9-week-old but not 6-week-old mice. On the other hand, in X–Y images of multi-channel AF, morphological changes of the spots were apparent at both time points.

Collagen network structures exist in the hepatic capsule, on the surface of the liver, which is about 20 µm thick. The structure of the hepatic capsule has not been extensively examined using conventional histopathological analysis. Moreover, it is difficult to visualize the entire shape of the structure by X–Y imaging using TPEM. In order to visualize and examine the collagen network in the hepatic capsule of NASH model mice, we reconstructed merged 3D images of SHG with a short-pass filter at 492 nm (cyan) and AF with emission filters at 500–550 nm (green) and 560–630 nm (red) (Fig. 2E, J, O, and T and Suppl. Fig. 2).

In the merged 3D images, the collagen fiber structures, which are indicated by the SHG signal (cyan), were located mainly on the surface of the liver (Fig. 2E, J, O, and T and Suppl. Fig. 2). In NASH model mice, the structure and texture of the collagen fibers of the hepatic capsule were heterogeneous at both 6 and 9 weeks, but no structural differences were detected between the two time points.

In addition, small spots of green (500–550 nm) or white color (500–550 nm and 560–630 nm) were observed in the merged 3D images (Fig. 2E, J, O, and T and Suppl. Fig. 2). These spots were larger and more heterogeneous in NASH model mice than in control mice at both 6 and 9 weeks, and in NASH model mice they were larger at 9 weeks than at 6 weeks. These results suggest that the size of these spots reflects the presence of disease, as well as the progression of fibrosis.

### 3.3 MIP-SHG imaging and quantitative evaluation

**Fig. 3:**
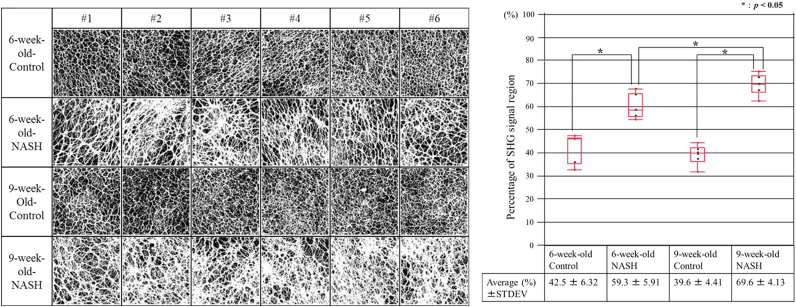
Binarized MIP-SHG images and boxplots of percentages of signal regions. (A) The analysis was carried out in six regions as shown in Suppl. Fig. 2 from control mice at 6 weeks (top panels), NASH model mice at 6 weeks (second panels), control mice at 9 weeks (third panels), and NASH model mice at 9 weeks (bottom panels). The MIP images from the SHG images of the indicated groups were binarized using ImageJ. (B) The reconstructed images in (A) were subjected to the quantitative evaluation in which the percentage of white area (indicating SHG signal) was measured as a ratio relative to the total number of pixels (512×512 pixels) in the acquisition area, and averaged for each group. *p<0.05 according to Student’s *t*-test.

As shown in Fig. 2E, J, O, and T and Suppl. Fig. 2, fibrillar collagen on the liver surface constituted only a single thin layer with a network-like structure that was almost perpendicular to the z-direction in our TPEM system. Besides, within the field of view we observed, the surfaces were not wavy, indicating low curvatures; thus, the signals along the z-axis fit into a narrow gap. These observations suggest that the structural information of the network is not lost in the z-axis projection of fibrillar collagen. Therefore, for simple and rapid quantitative evaluation, we decided to apply the MIP, which converts the Z-stack image sequence to a single-plane image by extracting the highest-intensity pixels along the z-axis in the SHG images (see Fig. 1). In order to obtain more effective characterization of fibrotic changes in the liver of NASH model mice, we binarized the MIP-SHG images, followed by quantification (Fig. 3). The fibrillar collagenous network was efficiently captured in specimens of 6- and 9-week-old control mice (Fig. 3A). The meshwork appeared to be distributed regularly, and the size was almost constant, suggesting that the control liver tissue maintained its normal state. On the other hand, in 6- and 9-week-old NASH model mice, fibrous collagen became broader and the area of the white region (SHG signal) became larger than in the control group. In addition, the spatial distribution of mesh sizes in the NASH group was heterogeneous. To quantify these binarized images, the ratio of the white (SHG signal) area to the total image area was calculated for each model. The results, depicted as boxplots in Fig. 3B, indicate that the average areas did not differ significantly between 6- and 9-week-old controls. The average areas of both 6- and 9-week-old NASH model mice were larger than those of the controls. Furthermore, 9-week-old NASH model mice had larger average areas than 6-week-olds. These findings suggest that liver fibrosis progresses with time in NASH model mice, consistent with the histopathological analysis.

### 3.4 MIP-AF imaging and quantitative evaluation

**Fig. 4:**
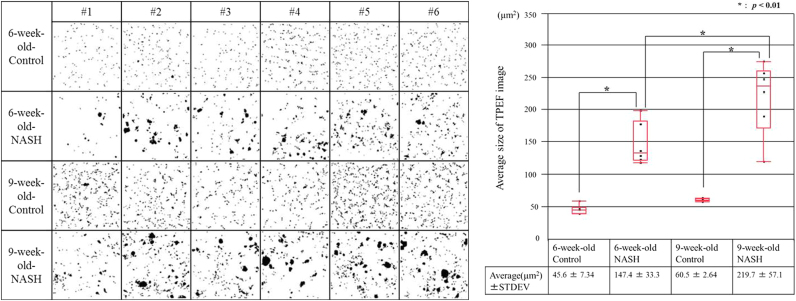
Binarized MIP-AF images and boxplots of average size. (A) The analysis was carried out six regions as shown in Suppl. Fig. 2 from control mice at 6 weeks (top panels), NASH model mice at 6 weeks (second panels), control mice at 9 weeks (third panels), and NASH model mice at 9 weeks (bottom panels). The MIP-AF images of the indicated groups were binarized using ImageJ. (B**)** Reconstructed images in (A) were subjected to quantitative evaluation in which the number of TPEF image spots (above 36 pixels) was counted and averaged for each group. *p<0.01 according to Student’s *t*-test.

We next analyzed AF images to quantitatively characterize the hepatic parenchyma of the liver tissue interior. As for the analysis of SHG images, we performed MIP of AF images (Fig. 4A). In contrast to the collagenous network observed in MIP-SHG images shown in Fig. 3A, the MIP-AF signals extended along a wide range of the z-axis. However, in the control group, the MIP-AF images contained a number of small spots. Independent, disconnected objects rarely overlap when an MIP is generated from a Z-stack. Therefore, information regarding object size distribution can be retrieved from the MIP images. After obtaining the MIP images, we performed binarization as was done for the SHG images. For both 6- and 9-week-old control mice, small objects were observed. In the NASH model mice, the number of spots was larger, and the spots comprised a mixture of small and large components. Calculations of object sizes (Fig. 4B) demonstrated that in the control group, these objects were smaller on average than in the NASH model groups. Moreover, the objects were significantly larger in 9-week-old NASH model mice than in 6-week-olds. These results are consistent with the results obtained from the analysis of SHG images.

## 4 Discussion

In this study, we developed a method for quantifying fibrotic and morphological changes in liver tissue using TPEM. This method is based on the acquisition of SHG and AF images. SHG imaging enables observation of non-centrosymmetric molecular assemblies such as fibrillar collagen in various organs without staining. The acquired signal reflects the localization and amount of fibrous structure of the collagen molecule, and enables us to detect quantitative changes in fibrosis, as well as fibrous degeneration, at microscopic resolution [9], [19], [21].

In this study, we first obtained 2D images of SHG and/or AF of liver tissues dissected from NASH model and control mice. As shown in Fig. 3, fibrotic and morphological differences between the two groups were observed at the advanced stage of NASH (9-week-old), but not at the early pre-NASH stage (6-week-old). We then reconstructed merged 3D images of SHG and AF from the same samples. As shown in Fig. 2E, J, O, and T and Suppl. Fig. 2, fibrotic and morphological differences were observed between NASH model and control mice. In particular, the structure and texture of the collagen fibers of the capsule on the surface of the liver were significantly perturbed and more heterogeneous in the model mice at the advanced stage of NASH. The structure of collagen network in the hepatic capsule has not been extensively visualized and examined by conventional histopathology; therefore, our method is a useful approach to evaluating fibrotic and morphological changes in the collage meshwork.

In order to evaluate SHG image, we used image processing by MIP in our study. This method is fast and easy to perform using available software. Our results demonstrate that the MIP-SHG method provides a unique and powerful tool for quantitative evaluation of fibrillar collagen deposits in this disease model.

Using our method, we quantified fibrillar collagen at the pre-NASH stage and progression of fibrosis from the pre-NASH to the NASH stage of the model (see Fig. 3). We could distinguish changes of fibrillar collagen deposits in hepatic capsule in the pre-NASH model from those in controls at 6 weeks (see Fig. 3B). At this stage, there was no histopathological finding of typical NASH. These results suggest that this method is useful for diagnosis of the early stage of the disease. In addition, changes in fibrillar collagen deposits could be also distinguished between the pre-NASH and the NASH stages, suggesting that this method is also useful for evaluating disease progression.

In addition, we also applied our method to AF imaging. As shown in Fig. 4A, MIP-AF (560–630 nm) imaging revealed size and morphological differences between the pre-NASH stage and the control. Moreover, differences were also apparent between the pre-NASH and NASH stages. These results suggest that MIP-AF imaging is very useful for diagnosis of the early stage of the disease, as well as evaluation of disease progression.

Notably in regard to the results described above, Wang et al. reported that spots in AF (350–650 nm) imaging indicate vitamin A in stellate cells of liver tissues [22]. In addition, activation of stellate cells is related with hepatitis [23]. Accordingly, a homogeneous distribution of small objects in AF (560–630 nm) images was observed in the control mice, whereas the spots became larger and morphologically more heterogeneous at the NASH stage (see Fig. 4A). These results suggest the possibility of activation of stellate cells and release of vitamin A from the cells during the early stages of hepatitis in the NASH model. The relationship between activated stellate cells, vitamin A release, and fibrillar collagen deposition in the capsule should be examined in future studies.
