# Supplementary material for: Quantitative imaging of fibrotic and morphological changes in liver of non-alcoholic steatohepatitis (NASH) model mice by second harmonic generation (SHG) and auto-fluorescence (AF) imaging using two-photon excitation microscopy (TPEM)
Source: Biochem Biophys Rep. 2016 Sep 25;8:277–83. doi: 10.1016/j.bbrep.2016.09.010 (PMC5614464; doi:10.1016/j.bbrep.2016.09.010)
Supplement: Supplementary file 2 — Supplementary material [file mmc2.docx]

**
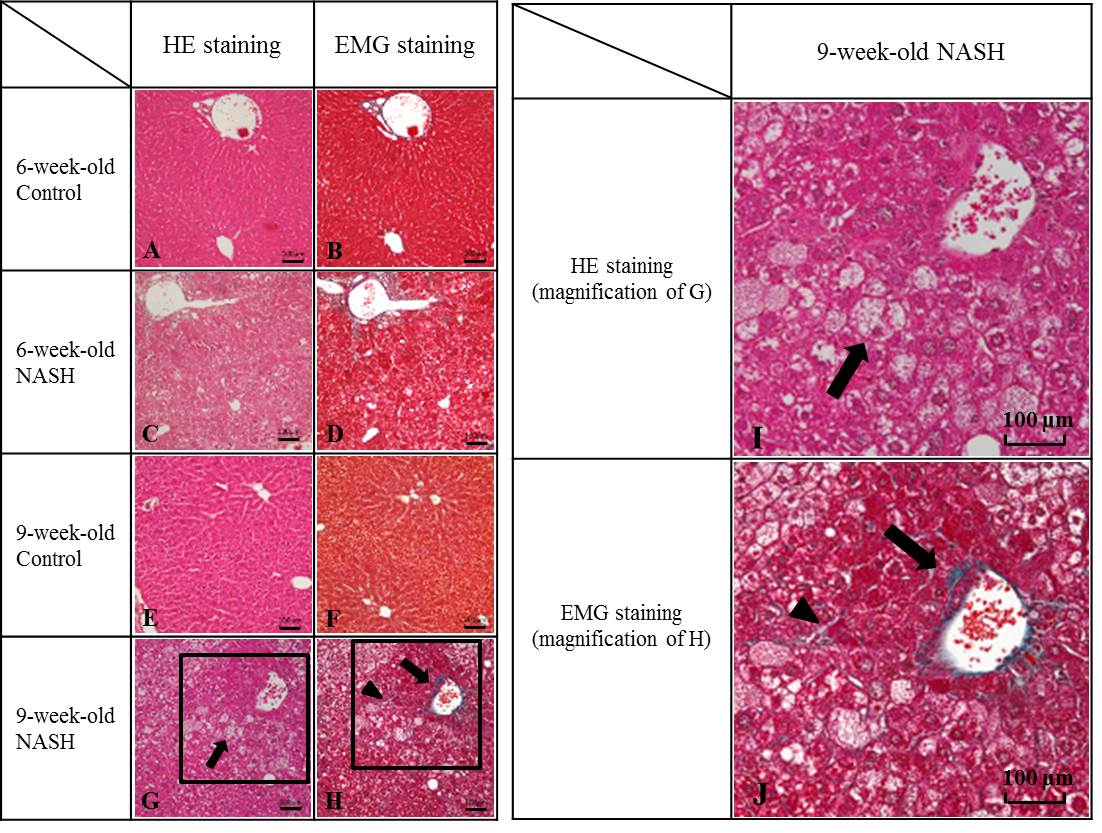
**

**Supplementary Figure 1. HE and EMG staining of the liver specimens.**

HE staining (A, C, E, and G) and EMG staining (B, D, F, and H) of liver tissue sections of the NASH model mouse at 6 weeks (C and D) and 9 weeks (G and H) and their respective controls (A, B and E, F). I and J are magnification of G and H, respectively. Black arrows in (G) and (I) indicate hepatocyte ballooning. Black arrows in (H) and (J) indicate fibrosis around the vessel. Black arrowheads in (H) and (J) indicate peripheral hepatocellular fibrosis.

**
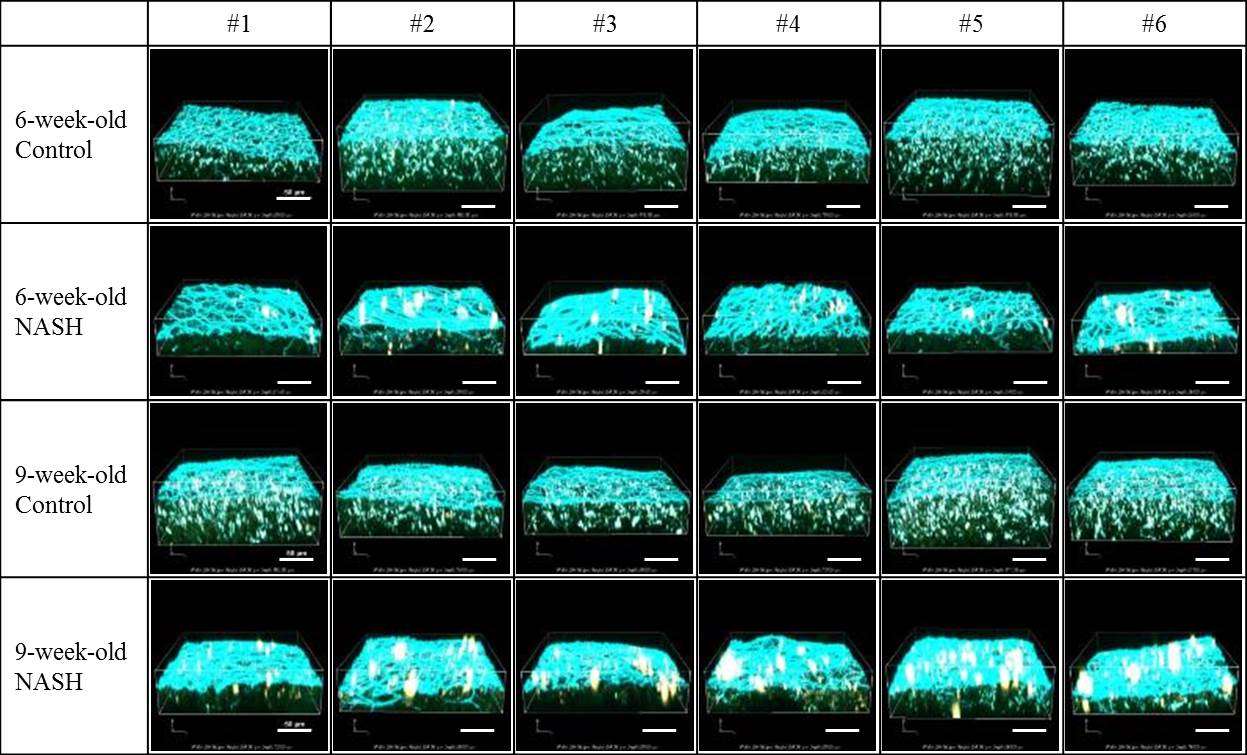
**

**Supplementary Figure 2. Three-dimensional reconstructed images of SHG and AF in liver tissues.**

The analysis was carried out in six regions from control mice at 6 weeks (top panels), NASH model mice at 6 weeks (second panels), control mice at 9 weeks (third panels), and NASH model at 9 weeks (bottom panels). Scale bars, 50 μm.
